# The Serotonin 5-HT_7_Dro Receptor Is Expressed in the Brain of Drosophila, and Is Essential for Normal Courtship and Mating

**DOI:** 10.1371/journal.pone.0020800

**Published:** 2011-06-02

**Authors:** Jaime Becnel, Oralee Johnson, Jiangnan Luo, Dick R. Nässel, Charles D. Nichols

**Affiliations:** 1 Department of Pharmacology and Experimental Therapeutics, Louisiana State University Health Sciences Center, New Orleans, Louisiana, United States of America; 2 Department of Zoology, Stockholm University, Stockholm, Sweden; University of Missouri, United States of America

## Abstract

The 5-HT_7_ receptor remains one of the less well characterized serotonin receptors. Although it has been demonstrated to be involved in the regulation of mood, sleep, and circadian rhythms, as well as relaxation of vascular smooth muscles in mammals, the precise mechanisms underlying these functions remain largely unknown. The fruit fly, *Drosophila melanogaster*, is an attractive model organism to study neuropharmacological, molecular, and behavioral processes that are largely conserved with mammals. Drosophila express a homolog of the mammalian 5-HT_7_ receptor, as well as homologs for the mammalian 5-HT_1A_, and 5-HT_2_, receptors. Each fly receptor couples to the same effector pathway as their mammalian counterpart and have been demonstrated to mediate similar behavioral responses. Here, we report on the expression and function of the 5-HT_7_Dro receptor in Drosophila. In the larval central nervous system, expression is detected postsynaptically in discreet cells and neuronal circuits. In the adult brain there is strong expression in all large-field R neurons that innervate the ellipsoid body, as well as in a small group of cells that cluster with the PDF-positive LNvs neurons that mediate circadian activity. Following both pharmacological and genetic approaches, we have found that 5-HT_7_Dro activity is essential for normal courtship and mating behaviors in the fly, where it appears to mediate levels of interest in both males and females. This is the first reported evidence of direct involvement of a particular serotonin receptor subtype in courtship and mating in the fly.

## Introduction

Serotonin (5-HT) is a monoamine neurotransmitter that regulates a variety of behaviors and physiological processes including circadian rhythms, sleep, appetite, aggression, locomotion, perception and sexual behavior in mammals [1], [2]. In mammals, there are fourteen different receptors than can be organized into seven families. The many effects of serotonin are primarily mediated through G-protein coupled receptors, which initiate multiple effector pathways [3]. Misregulation of serotonin signaling in humans has been implicated in neuropsychiatric disorders including depression, anxiety, anorexia nervosa, and schizophrenia.

In mammals, 5-HT_7_ mRNA has been observed in both the CNS and peripheral tissues including the suprachiasmatic nucleus of the hypothalamus, thalamus, hippocampus and cortex, as well as coronary artery, gastrointestinal tract, kidney, and spleen [3]. 5-HT_7_ receptors are expressed postsynaptically in the cortex, hippocampal formation and other parts of the brain [4]. They are, however, found both pre- and postsynaptically in the SCN [5]. Studies using antagonists and a knock out mouse model show involvement of 5-HT_7_ receptor activity in regulating mood, sleep, and circadian rhythms, as well as relaxation of vascular smooth muscles [6], [7], [8], [9], [10], [11], [12], [13]. With regard to sleep, 5-HT_7_ receptors modulate neuronal function in a number of areas of the brain that have been implicated in this behavior, including the SCN, DRN, thalamus and hippocampus [14]. The systemic administration of 5-HT_7_ receptor antagonist to rats at the beginning of light periods reduces total amount of REM sleep, and direct administration of antagonist into the DRN reduces REM sleep and the number of REM sleep periods [11]. Interestingly, 5-HT_7_ receptors have also been implicated in the regulation of mammalian sexual behavior. Activation of this receptor in rats mediates an inhibitory effect of female sexual behavior [15].

*Drosophila melanogaster* (the fruit fly) has proven to be a very effective model system for investigating the function of mammalian systems and diseases [16]. About 70% of human disease genes have functional orthologs in Drosophila [17], and the fly expresses functional orthologs of most mammalian neurotransmitter receptors, including receptors for dopamine, glutamate, acetylcholine, GABA, and serotonin, which mediate conserved behaviors [18]. The fruit fly expresses orthologs of three of the seven mammalian receptor families: 5-HT_1A/B_Dro, 5-HT_2_Dro and 5-HT_7_Dro, and the molecular pathways linking serotonin receptor interactions with behaviors are likely to be conserved between the two systems. 5-HT_1A/B_Dro are expressed in adult mushroom bodies, as well as additional brain circuits (unpublished data; [19]), and mediate aspects of sleep and aggression [19], [20]. We have previously characterized the 5-HT_2_Dro receptor, and found that it is expressed throughout the adult brain, including neurons within the protocerebrum and ellipsoid body, and mediates aspects of circadian behaviors and aggression [20], [21]. Here, we report on expression and function of the 5-HT_7_Dro receptor in *Drosophila melanogaster*. It is expressed within discreet circuits in the brain and ventral nerve cord, and is essential for normal courtship and mating.

## Methods

### Reagents

General laboratory chemicals and reagents were obtained from Sigma (St. Louis, MO). The 5-HT_7_ receptor antagonist SB258719 was obtained from TOCRIS, (Ellisville, MO).

### *Drosophila* strains and rearing

Fly strains obtained from other sources were Canton-S (CS), and UAS-mCD8::GFP (Bloomington Stock Center, Bloomington, IN). For routine maintenance, flies were reared on standard cornmeal-molasses food at 25°C under 12 hour light/dark conditions.

### Drug Administration in Courtship Assay

For the mating assays, bottles of wild type CS flies were cleared and newly eclosed, virgin females and males were collected and matured for 5-6 days prior to testing in 15 mL conical tubes containing ∼300 µL of food (10% sucrose, 1% agarose and the appropriate drug) and plugged with cotton at the open end. In the courtship and mating assays, flies were maintained on food + drug for 5 days to ensure that steady-state levels were reached. To determine if the presence of SB258719 affected the feeding behaviors of the flies, a CAFÉ assay was performed following established protocols [22] and feeding a 10% sucrose solution with or without 3 mM SB258719. No statistical differences were observed in the feeding behaviors between the two groups of flies over five days (data not shown).

### Courtship and Mating Assay

Between five and six virgin females were housed together during this process, while sexually naïve males were individually housed. During the maturation period, all flies were maintained at 25°C under a 12 hour light/dark cycle until testing. Following the maturation period, one male and one female were transferred to a single chamber of a mating wheel. The mating wheel is a circular piece of 1.0 cm thick plexiglass 10.0 cm in diameter with ten circular chambers are drilled into the wheel at the outer edge, approximately 1.0 cm in diameter and 5.0 mm deep. A second circular piece of 2.0 mm plexiglass that is able to rotate freely is attached to the lower plexiglass wheel and serves as a cover for the mating chambers. A single 3.0 mm hole in the top is used to insert flies into the chambers. Our mating chambers are slightly larger than those used by Ejima and Griffith [23], but are consistent with other chambers used in published reports [24]. Heterosexual courtship in *Drosophila melanogaster* involves a progression of behaviors occurring in a defined order: orientation of the male toward the female, tapping, wing song, licking of the female genitalia, and curling of the male (attempted copulation), with successful copulation occurring shortly thereafter [25], [26]. Each mating pair was closely monitored for 10 minutes and scored for latency in performing orientation, wing vibration, licking, curling and copulation. The frequency that the behaviors occurred (number of pairs successfully performing a behavior out of the total number of pairs tested), as well as the duration of copulation were also determined. The number of copulation attempts, as well as the duration of the copulation were also recorded. Flies that successfully copulated within the initial 10-minute observation period were monitored until completion of copulation or for a total of 20 minutes. If no copulation occurred within the first 10 minutes, the pairs were observed for up to 60 minutes, but only for successful copulation within this time. All testing was performed at 25°C at 70–80% relative humidity, and between the hours of 11 am and 4 pm.

### Odor Avoidance

Between 100 and 150 1–3 day old CS flies were collected and maintained on standard food with or without 5-HT_7_ antagonist (3.0 mM SB258719) for 48 hrs prior to testing for olfactory avoidance in a large 64 ounce commercial juice bottle with the large end cut off and replaced with fine plastic mesh. Flies were then transferred to the choice point of t-maze device (a standard olfactory learning and memory apparatus), where they were presented with an aversive odor (either 3-methylcyclohexanol or benzaldehyde, at varying concentrations) in one arm of the apparatus paired with fresh room air in the opposite arm of the apparatus for 120 seconds following established protocols used in olfactory learning and memory assays [27]. Performance indices are calculated as the number of flies avoiding the aversive odor minus the number of flies that do not avoid the aversive odor, and the difference divided by the total number of flies tested. All experiments were performed at 70–80% relative humidity and 25°C.

### Locomotion Assay

Male flies were collected less than 72 hours post eclosion and anesthetization on ice. Individual flies were then placed into 5 mm diameter glass capillary tubes with an agar plug at one end consisting of 1% agarose, 10% sucrose and 3 mM drug (where appropriate), and then plugged at the other end with cotton. Tubes were then placed into Trikinetics (Waltham, MA) activity monitor arrays, which were subsequently placed into a humidified incubator at 25°C with a 12 h light-dark cycle. Infrared beam breaks, as a measure of activity, were monitored with the Trikinetics Drosophila Activity Monitor System (DAMS). Sixteen males were used in each experiment for each treatment and monitored for seven days. Only activity data for days 3 and greater were used for analysis, omitting the first two days to allow for acclimation to the environment and to build up steady state drug levels.

### Generation of the 5-HT_7_Dro GAL4 expression construct and transgenic lines

#### Preparation of 5-HT_7_Dro promoter region

Genomic DNA was prepared by homogenizing 25 wild type Oregon-R flies in 400 ml lysis buffer (30 mM Tris (pH 9), 100 mM EDTA, 0.6% SDS, 0.5% sucrose) followed by heat inactivation for 15 min at 70°C; proteins were precipitated out by addition of 80 ml 6M KOAc on ice for 30 minutes followed by centrifugation at 4°C at maximum speed in a microcentrifuge. The aqueous supernatant was extracted with an equal volume of phenol, phenol-chloroform, then chloroform. Nucleotides was precipitated by addition of 2 volumes of ethanol, incubation at room temperature for 5 minutes, and centrifugation for 10 minutes in a microcentrifuge at room temperature. The pellet was washed in 75% ethanol, resuspended in 200 ml TE buffer +1 ml RNAse (Epicentre, Madison, WI), and incubated at 37°C for one hour. DNA was precipitated by addition of 0.1 volumes of 3M NaOAc, 2.5 volumes of ethanol, incubation at –20°C overnight, and centrifugation for 15 minutes at maximum speed in a microcentrifuge at 4°C. The pellet was washed with 75% ethanol, allowed to air dry for 5 minutes, and resuspended in 25 ml sterile H_2_O.

**Figure 1:**
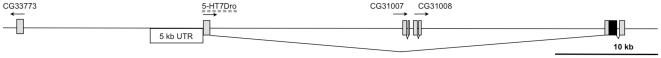
Map of 5-HT_7_Dro region. The 5-HT_7_Dro locus on the third chromosome is approximately 40 kb with a large first intron that contains two additional short annotated transcripts. The 5 kb region of genomic DNA immediately upstream of the mRNA transcript start site used to generate the 5-HT_7_Dro GAL4 strain is shown (white box). Grey boxes indicate untranslated regions of exons, darker boxes represent translated regions. Arrows indicate direction of transcription. Scale bar is shown.

To isolate putative 5′ enhancer regions, which are normally contained within the first few kb of genomic DNA upstream of the RNA transcription start site, 5 kb of genomic DNA immediately upstream of the ATG start codon within the 5-HT_7_Dro locus was amplified from 1 µl of genomic DNA using the Expand High Fidelity PCR System from Roche (Indianapolis, IN) following manufacturers instructions (Figure 1). Primers corresponding to the 5-HT_7_Dro promoter region containing Not I restriction sites at their 5′ end were ordered from Integrated DNA Technologies (Coralville, IA). Forward primer = 5′-gcggccgcGGTAGCCAAATGAACGTTGAGCGC-3′; Reverse Primer  = 5′- gcggccgcACGAATCGAATATCTGAATTCCGC-3′; annealing T = 55.0°C, elongation T = 68°C. The amplification product consisted of a single band of 5 kb, which was gel purified using the Zymo Gel DNA Recovery Kit (Orange, CA) following manufacturers instructions.

#### Construction of pERGP GAL4 expression vector

The pCaSpeR4 plasmid (Dr. Bih-Hwa Sheih, Vanderbilt University, Nashville, TN) was digested with Kpn I (Promega, Madison, WI) and blunt ended using the End-IT DNA End Repair Kit (Epicentre, Madison, WI). The GAL4-hsp70 fragment from the pGaTB vector (Dr. Norbert Perrimon, Harvard University, Boston, MA) was excised by digesting with Not I and Bam HI (Promega), followed by gel purification, and blunt ending. The GAL4-hsp70 fragment was ligated into the blunt ended Kpn I cut pCaSpeR4 vector using the Fast-Link DNA Ligation Kit from Epicentre following manufacturers directions. The resulting product, pERGP (‘Enhancer-Ready Gal4-P element’), contains a unique Not I restriction site 5′ of the GAL4 region, and a unique Eco RI restriction site 3′ of the hsp70 terminator region for the subcloning of enhancer elements into either, or both, unique restriction sites. The 3′ Eco RI site may be useful for inclusion of intronic enhancers when generating GAL4 expression constructs.

#### Generation of the 5-HT_7_Dro construct and transgenics

Both the purified 5-HT_7_Dro PCR product and pERGP were digested with Not I and gel purified. Digested pERGP was dephosphorylated using Apex Heat-Labile Alkaline Phosphatase (Epicentre) following manufacturers directions. The 5-HT_7_Dro promoter fragment was ligated into the Not I site of the pERGP vector using the Fast-Link DNA Ligation Kit following manufacturers directions. The final construct was verified using a panel of restriction enzymes, as well by sequence analysis of the cloning site junctions. Five independent transgenic lines were generated from this final product using the services of BestGene Inc. (Chino Hills, CA).

### Generation of the sym-p5-HT_7_-RNAi construct and transgenic lines

To generate the sym-p5-HT_7_RNAi plasmid, we used the full length 5-HT_7_Dro cDNA (DGRC #4507) as template for a PCR reaction with the forward primer = 5′-ataagaattcCGCAGGACTTTAATAGCAGTAGC -3′ (with the restriction sequence for Eco RI added to the 5′ end of the primer) and the reverse primer = 5′-CTTCTCTTTGGCCAGTTGA - 3′ (Integrated DNA Technologies) using the Expand High Fidelity kit from Roche. The PCR product was digested with BglII and EcoRI (Promega), and the 800 bp fragment was gel purified using the Zymo Gel DNA Recovery Kit (Orange, CA) per the manufacturers instructions.

The sym-pUAST vector contains two regions, each containing five UAS activating sequences in opposite orientations [28] (Gift of Dr. Wendi Neckameyer, St. Louis School of Medicine, St. Louis, MO). The vector was digested with BglII and EcoRI (Promega), and gel purified. Digested sym-pUAST was then dephosphorylated using Apex Heat-Labile Alkaline Phosphatase (Epicentre) following manufacturers directions.

The prepared 5-HT_7_Dro cDNA fragment was ligated into the sym-pUAST vector using the Fast-Link DNA Ligation Kit following manufacturers directions. The construct was verified using a panel of restriction enzymes, and by sequence analysis of the cloning site junctions. Independent transgenic lines were generated from this final product using the services of BestGene Inc. (Chino Hills, CA).

QPCR was performed on the RNA isolated from the heads of F1 crosses and their parentals between the 5-HT_7_Dro-GAL4 and sym-pUAST-5-HT_7_Dro flies to determine knockdown efficiency. Total RNA from 20 combined heads was isolated by Tri-Reagent (Molecular Research Center, Cincinnati, OH) following manufacturers protocols. First strand cDNA synthesis was performed using the Improm-II kit from Promega using 0.3 µg total RNA per reaction following manufactures directions using random hexamer primers. Quantitative real-time PCR assays were designed using the Universal ProbeLibrary system (Roche, Indianapolis, IN; https://www.roche-applied-science.com). Amplicon primers and universal probes utilized for the 5-HT_7_Dro mRNA and the reference standard, *ribosomal protein L32* (*RpL32*) mRNA were: RpL32 (U#105) F: 5′-CGGATCGATATGCTAAGCTGT-3′, R: 5′--GCGCTTGTTCGATCCGTA-3′; 5-HT_7_Dro (U#44) F: 5′-AATGATTCTGAGGCTCGAAGA-3′, R: 5′ TATGAGCAACCCAGTGCTGA-3′ QPCR was performed with the BioRad iCycler IQ5 (BioRad, Hercules, CA) using the HotStart-IT Probe qPCR Master Mix (USB, Cleveland, OH) following the manufacturers instructions (25 µl reaction volume; cycle parameters: initial 95°C for 2 min, followed by 44 cycles of 95°C 15 sec, 60°C 45 sec) in 96 well plate format. Reactions were performed in quadruplicate for each gene and genotype. Expression of *RpL32* was used as the reference control to normalize expression between genotypes. Expression levels were calculated using the ΔΔC_T_ method (ABI: User Bulletin #2, ABI Prism 7700 Sequence Detection System, 10/2001).

### Immunohistochemistry

Larva and adult brains were dissected in 0.1 M sodium phosphate buffer (pH 7.4) and fixed in PLP (2% paraformaldehyde, 75 mM lysine, 10 mM sodium periodate, pH 7.4) for 90 mins, permeabilized in 0.1 M sodium phosphate buffer (pH 7.4), with 0.1% saponin and 0.4% NP40 for 30 mins at room temperature, followed by incubation overnight at 4°C with primary antibody in 0.1 M sodium phosphate buffer (pH 7.4), with 0.1% saponin and 0.4% NP40. The primary antibodies used were rabbit-anti-5-HT (1∶750; Sigma), and mouse-anti-PDF (1∶50; Developmental Studies Hybridoma Bank, Iowa City, Iowa). After three washes in 0.1 M sodium phosphate buffer (pH 7.4), tissues were incubated in secondary antibody for one hour at room temperature. Secondary antibodies used were mouse Alexa 633 conjugated anti-rabbit (1∶750) (Invitrogen), and goat Texas Red conjugated anti-mouse (1∶150) (Santa Cruz Biotechnology, Santa Cruz, CA). After staining, brains were washed in 0.1 M sodium phosphate buffer for 3×20 min, then cleared through a series of glycerol (25%, 50%, 75%, 90%), and mounted in 90% glycerol. To visualize the 5-HT_7_Dro circuitry in 5-HT_7_Dro-gal4/UAS-mCD8::GFP flies, as well as conjugated secondary antibodies, optical sections of whole brains were acquired on a Leica TCS-SP2 confocal microscope (Leica Microsystems, Exton, PA, USA) at a thickness of 0.25–0.5 µm. For the detailed analysis of serotonin, fruitless and 5-HT_7_Dro-GAL4 expression in the central adult brain and ventral nerve cord, 5-HT_7_Dro-gal4/UAS-mCD8::GFP tissues were fixed in 4% paraformaldehyde in 0.1 M sodium phosphate buffer. After thorough washes in buffer, dissected brains were incubated in a mouse monoclonal antibody to serotonin (Clone 5HT-H209; Dako, Copenhagen, Denmark) at dilution of 1∶80. For visualization of the male form of the Fruitless protein, Fru^M^, expression we utilized a rabbit antiserum to Fru^M^ [29], kindly provided by Dr S. F. Goodwin (Univ. Oxford, UK). This antiserum was applied at a dilution of 1∶2.500 and detected by an Alexa 546-tagged secondary antibody (Invitrogen) diluted to 1∶1000. To visualize the serotonin antiserum, Cy2 conjugated goat anti-mouse antiserum (Jackson Immuno Research) was used at a dilution of 1∶1500. To visualize the 5-HT_7_Dro circuitry, 5-HT_7_Dro-GAL4/UAS-mCD8::GFP flies were used. Confocal images were collected on a Zeiss laser scanning microscope (LSM 510 META) based on an Zeiss Axiovert S100 microscope with an Argon2/488 nm and HeNe 543 nm lasers. Images were obtained at an optical section thickness of 0.5–0.9 µm, assembled in the Zeiss LSM software and were edited for contrast and brightness in Adobe Photoshop CS3 Extended version 10.0.

## Results

### The 5-HT_7_Dro receptor is expressed in the larval and adult brain

**Figure 2:**
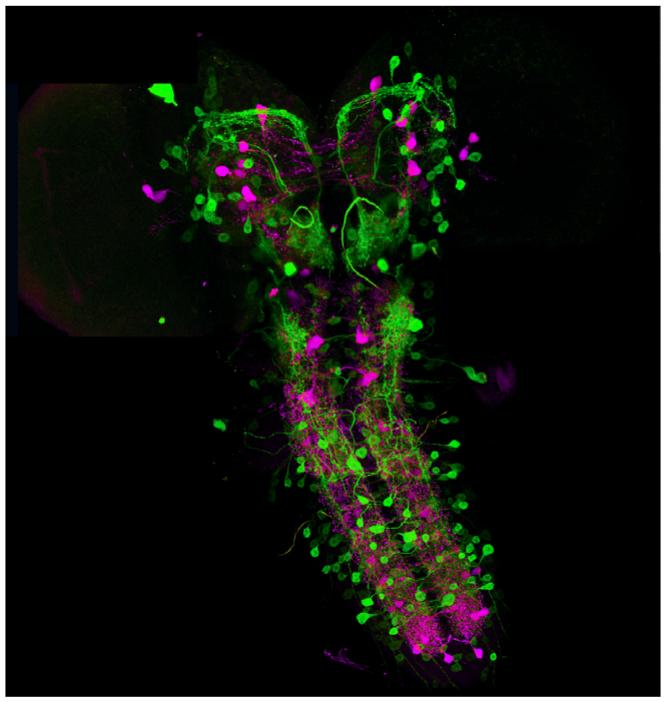
5-HT_7_Dro-GAL4 expression in the brain of third instar larva. 5-HT_7_Dro-GAL4 driven expression of GFP (green) is detected in distinct circuits within the brain of wandering third instar larva as well as in the ventral ganglia. Antisera to 5-HT detected with secondary antibodies conjugated to alexafluor 568 (magenta) highlights the presynaptic serotonergic circuitry. No overlap indicates that 5-HT_7_Dro-GAL4 expression is postsynaptic.

Heterozygous flies carrying both the 5-HT_7_Dro-GAL4 and UAS-mCD8::GFP constructs were used to examine the expression patterns of the 5-HT_7_Dro-GAL4 element. GFP expression, representing putative 5-HT_7_Dro expression, is observed in discreet populations of cells in the hemispheres of the 3^rd^ instar larva brain, as well as in neurons in the ventral ganglia (Figure 2). Two independent insertion lines produced identical expression patterns, two produced no expression, and one strain was lost before expressing testing could be completed. The parental homozygous UAS-mCD8::GFP responder strains did not demonstrate any detectable expression (data not shown). To determine if 5-HT_7_Dro expression is pre- or post-synaptic, immunohistochemistry was performed with anti-serotonin antibodies. 5-HT_7_Dro-GFP expression in the larvae brain does not co-localize with anti-5-HT immunoreactivity (Figure 2), indicating postsynaptic expression in the larva.

**Figure 3:**
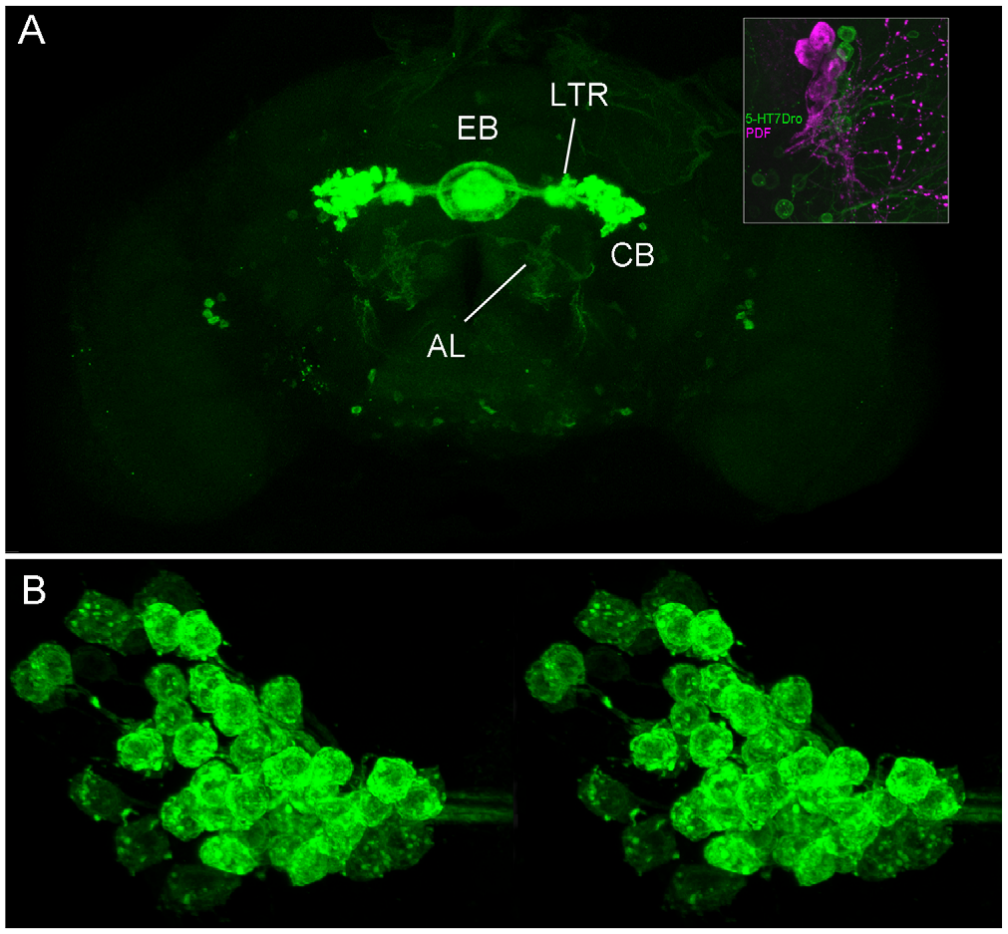
5-HT_7_Dro-GAL4 expression in the adult brain. A) Expression of the 5-HT_7_Dro-GAL4 driver is highly localized to large field R-neurons of the ellipsoid body. There are additional groups of cells that cluster with, but do not express peptide dispersal factor (PDF, magenta), between the central brain and the optic lobes (inset). B) High resolution cross-eyed stereo view of a typical cluster of large field R-neurons expressing 5-HT_7_Dro-GAL4. This particular cluster consists of 48 large-field R neurons that express the driver. (EB ellipsoid body; LTR  =  lateral triangle, CB  =  cell bodies; ATL =  antennal lobe).

In the adult brain, 5-HT_7_Dro-GFP is expressed at high levels in large-field R neurons that innervate the ellipsoid body, and within discreet populations of cells between the central brain and the optic lobes (Figure 3A). The location of the small cluster of 5-HT_7_Dro-mCD8 expressing cells between the central brain and the optic lobe are reminiscent of the location of PDF (peptide dispersing factor)-expressing sLNvs and lLNvs described in Helfrich-Forster [30] that are members of the circadian clock circuitry. To determine if these are indeed PDF-expressing clock neurons, we co-stained with anti-PDF antibodies, and revealed that whereas 5-HT_7_Dro-GAL4 expressing neurons are not the PDF neurons, they tightly cluster with the PDF-expressing cells (Figure 3A inset). This clustering suggests that 5-HT_7_Dro neurons may modulate or influence the function of the PDF-positive LNvs, and may have a role in circadian behaviors. A close inspection of the cluster of large-field R neurons within the central complex reveals 5-HT_7_Dro-GAL4 expression in greater than 40 large neurons of each cluster (Figure 3B). Their neurites run in a tract to the lateral triangle and thereafter to the ellipsoid body. Previously described ring neurons have their cell bodies in this area [31], [32], [33]. Interestingly, as exemplified by Figure 3B, we detect more large-field R neurons per cluster than previously reported (∼48 per cluster). Previous studies utilizing GAL4 drivers to highlight various subpopulations of large-field R neurons have only indicated the presence of 38–40 neurons per cluster [31]. Our results suggest that not only does the 5-HT_7_Dro-GAL4 driver likely express in all known large-field R-neurons, but that there may be additional yet to be defined subpopulations of neurons within the clusters. Very weak 5-HT_7_Dro-Gal4 expression in the upper region of the fan-shaped body, but not in other substructures of the central complex, was also detected (not shown).

**Figure 4:**
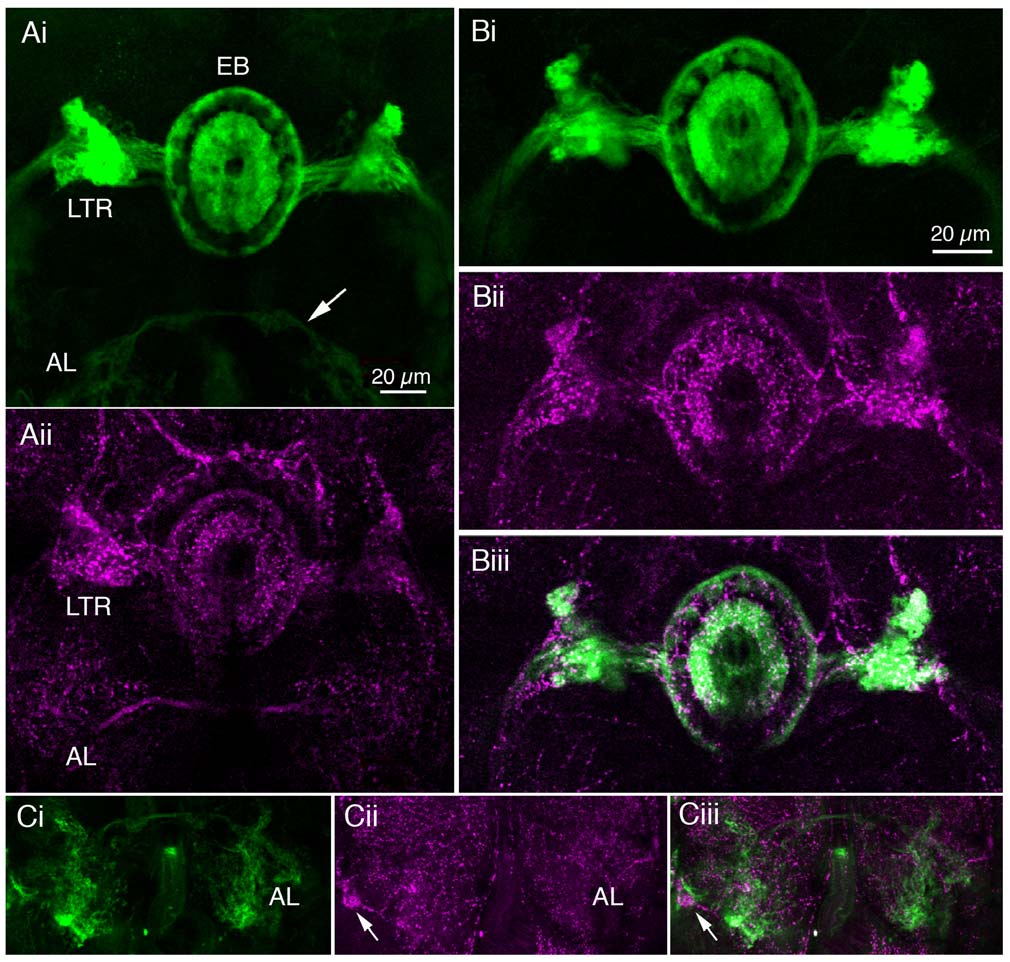
Distribution of 5-HT_7_Dro-GAL4 expression and serotonin in adult Drosophila brain. Ai) Within the ellipsoid body (EB) of the central complex, the innermost and outermost rings display 5-HT_7_Dro-GAL4 expression (green). The lateral triangles (LTR) of the central complex also display GFP. Expression is also seen more ventrally in antennal lobe neurons. Aii) Serotonin-immunoreactive neuron processes highlight the presence of 5-HT in the innermost and outermost rings of the EB as well as the lateral triangles and antennal lobes (magenta). Bi) Close up view of 5-HT_7_Dro-GAL4 expression in the central EB. Bii) The same field as in Bi showing serotonin-immunoreactive neuron processes (magenta). Biii) Merge of Bi and Bii. Note the prominent superposition of receptor and serotonin distribution in the two rings of the EB and in the LTR. Ci-Ciii) Distribution of 5-HT_7_Dro-GAL4 expression and serotonin-immunoreactivity in antennal lobes (AL). The receptor is seen in select glomeruli of the lobes (Ci), whereas serotonin is distributed in varicose processes throughout the lobes (Cii). The arrow indicates the cell body of the left serotonergic antennal lobe interneuron. The merged channels are seen in Ciii.

We performed serotonin-immunolabeling on brains of adult flies expressing 5-HT_7_Dro-GAL4 driven GFP to study spatial relations between ligand and receptor. Whereas the distribution of serotonin-immunoreactive neuronal processes is abundant throughout most of the major brain neuropils, 5-HT_7_Dro-GAL4 mediated GFP expression is most prominent in part of the central complex and in the antennal lobe (Figure 4Ai). In the central complex serotonin-immunoreactive processes can be seen in the fan-shaped body and ellipsoid body, as well as in the lateral triangles (Figure 4Aii). Both serotonin-immunolabeling and 5-HT_7_Dro-GAL4 expression is seen in the anterior and posterior rings of the ellipsoid body and in the lateral triangles (Figure 4). The neural processes seen with the two markers are clearly superimposed in these regions (Figure 4Biii). Again, the receptor appears to be postsynaptic, since the R-neurons do not display serotonin-immunoreactivity (Figure S1).

Two large centrifugal neurons with processes in most of the glomeruli of the antennal lobe are known to produce serotonin [34], [35]. Comparing serotonin immunolabeling with 5-HT_7_Dro-GAL4 expression, GFP is observed in select glomeruli, whereas the serotonergic neurons arborize in most, if not all (Fig. 4C). As seen in Fig S1 B the large cell bodies of the serotonergic antennal lobe neurons do not coexpress 5-HT_7_Dro-GAL4. This GAL4 expression in the antennal lobes is weak, but enhancement of the GFP signal reveals a large interneuron in each hemisphere that supply branches to the glomeruli that is distinct from the serotonergic interneuron (Fig. S1 C, D). In general, serotonergic processes are far more abundantly distributed than those of the 5-HT_7_Dro-GAL4 expressing neurons, suggesting that serotonin acts via other 5-HT receptors in most parts of the brain.

**Figure 5:**
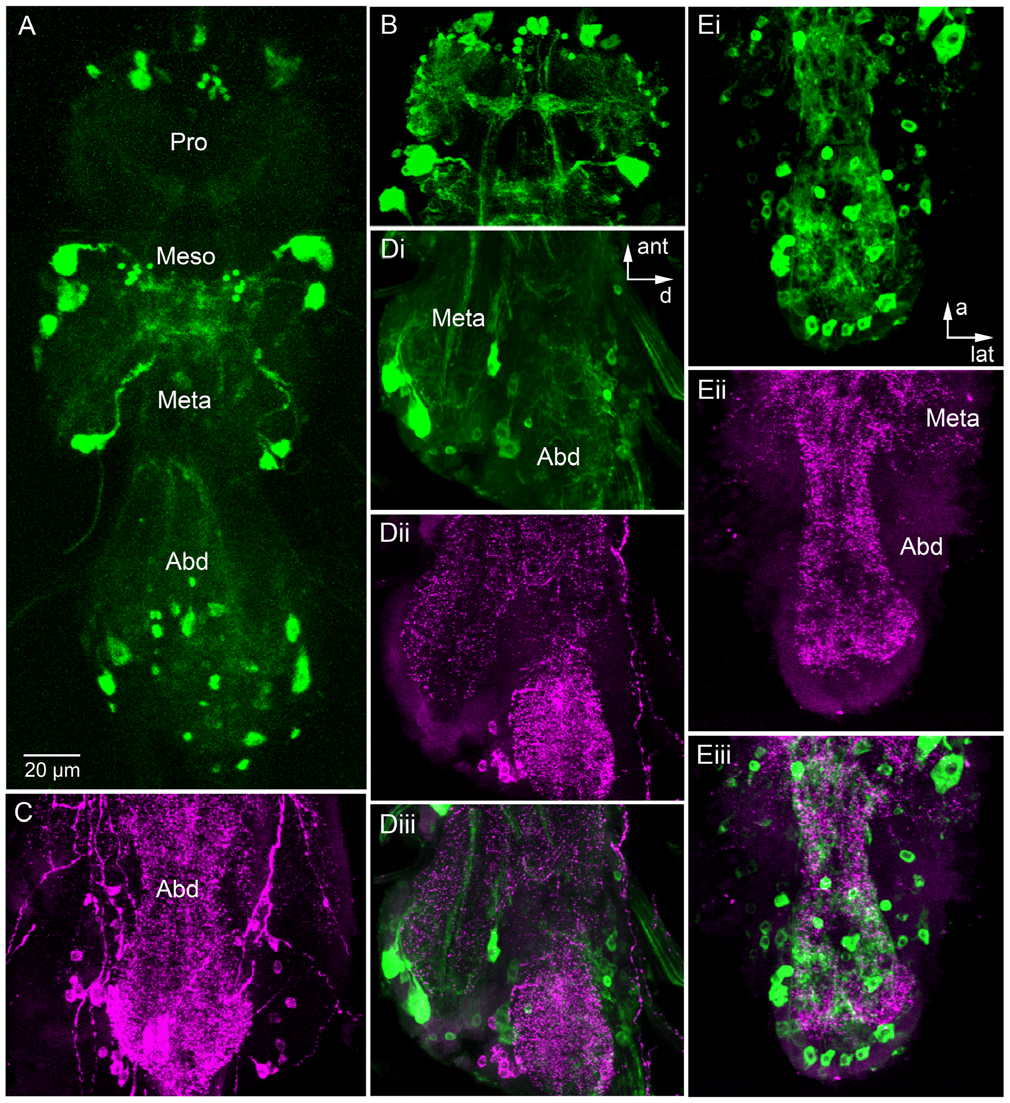
Distribution of 5-HT_7_Dro-GAL4 expression and serotonin in adult Drosophila ventral nerve cord. Anterior is up in all panels and scale bar applies to all. GFP-expression is shown in green and serotonin-immunolabeling in magenta. A) Overview of ventral nerve cord with segmental distribution of 5-HT_7_Dro-GAL4-expressing neurons in pro-, meso- and metathoracic and abdominal (Abd) neuromeres. Note the large lateral cell bodies in meso- and metathoracic neuromeres. B) Different optical section plane of pro- and anterior mesothoracic neuromeres. Note tracts of GFP-labeled neuronal processes. C) Very dense distribution of serotonin-immunoreactive processes in abdominal neuromeres. Di-iii) A sagital view of the metathoracic and abdominal neuromeres with receptor and serotonin distribution. Note that markers are not colocalized in any neuronal structures, suggesting postsynaptic distribution of the 5-HT_7_Dro. However, processes of the two types of neurons superimpose in neuropil regions. At arrows: ant, anterior and d, dorsal. Ei-iii) Horizontal views of the same neuromeres. No colocalized markers can be detected, but overlap between fibers. At arrows: a, anterior and lat, lateral.

Expression of 5-HT_7_Dro-GAL4 is also detected in several neurons in the ventral ganglion of the adult. The Gal4-driven GFP was seen in paired neurons in all thoracic and abdominal neuromeres of the ganglion (Fig. 5A, B). In each of the meso and metathoracic neuromeres there are two pair of neurons with large lateral cell bodies displaying GFP (Fig. 5A, Di). Neurons with smaller cell bodies were detected more medially in each neuromere. In abdominal neuromeres larger and smaller 5-HT_7_Dro-expressing cell bodies were distributed without a strict segmental organization (Fig. 5A, E). Altogether we could detect approximately 40 neuronal cell bodies in the thoracic neuromeres and about 30 in the abdominal. GFP labeled neuronal processes were seen in neuropil of the different neuromeres, some were organized in distinct tracts (Fig. 5B). By applying antiserum to serotonin to 5-HT_7_Dro-GAL4-GFP expressing ganglia we could show that the two markers are not colocalized in any neuron (Fig. 5D, E), suggesting post-synaptic distribution of the receptor. The serotonin-immunoreactive processes are very abundant in thoracic and abdominal neuropils (Fig. 5C) and double labeling revealed superposition of these and the branches of 5-HT_7_Dro-GAL4-expressing neurons (Fig. 5D, E). Because of the potential role of the receptor in courtship, as described next, we also examined 5-HT_7_Dro-GAL4 expression in relation to the male form of Fruitless (Fru^M^), a key protein involved in sexual behaviors. We did not detect any colocalization between 5-HT_7_-GAL4 and Fru^M^ expression in the ventral nerve cord of adult flies (Figure S2). This is the region where it was shown previously that a small population of abdominal serotoninergic neurons co-express Fru^M^ [36], [37].

### 5-HT_7_ receptor activity is essential for normal courtship and mating behavior

In Drosophila, complex behaviors such as learning and memory, aggression, locomotor reactivity, circadian rhythm, olfaction, and sleep are mediated by higher brain centers such as the mushroom body, ellipsoid body, and central complex [30], [38], [39]. The mushroom body has been implicated in Drosophila courtship and mating behavior [40], [41], [42], however, the precise role of the ellipsoid body, and other components of the central complex has yet to be explored.

**Figure 6:**
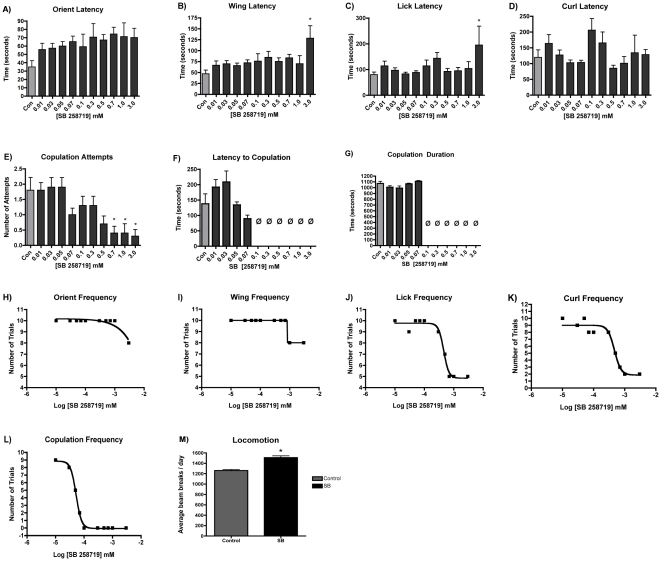
5-HT_7_Dro modulates courtship and mating. Mating pairs fed increasing amounts of the antagonist SB285719 (black bars) were observed for ten minutes to assay the latency of orient, wing vibration, licking, and curling (A, B, C, D, F), the number of copulation attempts (E), the duration of copulation (G), and the frequency at which each behavior occurs (H-L). The latencies of SB-treated flies performing a specific behavior did not differ significantly from untreated controls (gray bars) with increasing drug doses (A, B, C, D, F) with the exception of curling and licking at 3 mM. The number or copulation attempts (E) were significantly decreased at doses above 0.7 mM, while the frequency of attempted copulations decreased with doses above 0.05 mM. Whereas flies fed doses of SB greater than 0.07 mM did not successfully copulate (F, L), the duration of successful copulations at lower doses did not differ from controls (G). (n = 10 pairs observed for each behavior; * p<0.01, ANOVA with Dunnett's post hoc test for multiple comparison; φ = No successful copulation). M) Wild-type OR male flies were loaded into the DAMS monitoring system and maintained on 10% sucrose, 1% agarose (gray bar) or the same medium supplemented with 3 mM SB (Black bars). Locomotion was measured by counting the total number of beam breaks in a 24 hour period. Number of beam breaks per hour were counted and averaged over a three-day period. SB-treated flies exhibited only a slight increase in levels of activity compared to control flies.

The effects of the 5-HT_7_ receptor antagonist, SB258719 (SB), on courtship behavior were assessed after maintaining flies for five days on food containing various amounts of drug ranging from 0.01 mM to 3.0 mM (Figures 6 A-L). SB258719 is approximately 100 fold more selective for the 5-HT_7_ receptor than other 5-HT receptors in mammals [43], however the specificity for the drug at Drosophila receptors has not yet been defined. Courtship pairs were assayed for orient latency, wing vibration latency, lick latency, curl latency, copulation attempts, copulation latency, and copulation duration, as well as frequencies for each behavior. Whereas the frequencies of behaviors were observed to generally decrease with increasing drug, the latencies of flies still performing a particular behavior at any given drug level were not significantly different from that of controls with the exception of wing song and licking latencies at 3.0 mM SB, which were about double that of control (Figure 6 B, C). All pairs for a given treatment exhibited normal orienting behavior except for pairs fed 3.0 mM SB, where only 8 of 10 pairs exhibited this behavior (Figure 6H). All pairs also demonstrated wing vibration at all drug levels except for 1.0 and 3.0 mM SB fed pairs, where only 8 of 10 pairs exhibited this behavior (Figure 6I). These relatively normal early behaviors of courtship in flies fed higher levels of SB indicate that sensory processes are not likely being disrupted by the SB antagonist. If they were, the fly would not be expected to perform normally behaviors that rely upon visual, auditory, taste, or olfactory sensory cues.

Decreases in licking frequency were observed at levels greater than 0.5 mM SB, with an IC_50_ = 0.47 mM (Figure 6J). The number of copulation attempts were significantly reduced in pairs treated with 0.7, 1.0, and 3.0 mM drug (Figure 6E), and the frequency at which this behavior occurred decreased at drug concentrations greater than 0.05 mM with an IC_50_ = 0.47 mM (Figure 6L). Remarkably, only flies fed drug at or below 0.07 mM successfully copulated, with an IC_50_ = 0.05 mM (Figure 6L). The latencies (Figure 6F) and durations (Figure 6G) of the few pairs of flies that successfully copulated were not significantly different from that of controls, however, these behaviors were observed in fewer pairs as drug concentrations were increased (Figure 6L). To determine if the observed effects of the SB drug were simply due to drug-induced decreases in activity levels, general activity was measured using the Trikinetics DAMS system. SB treated flies were observed to be only slightly more active than untreated control flies (Figure 6M).

**Figure 7:**
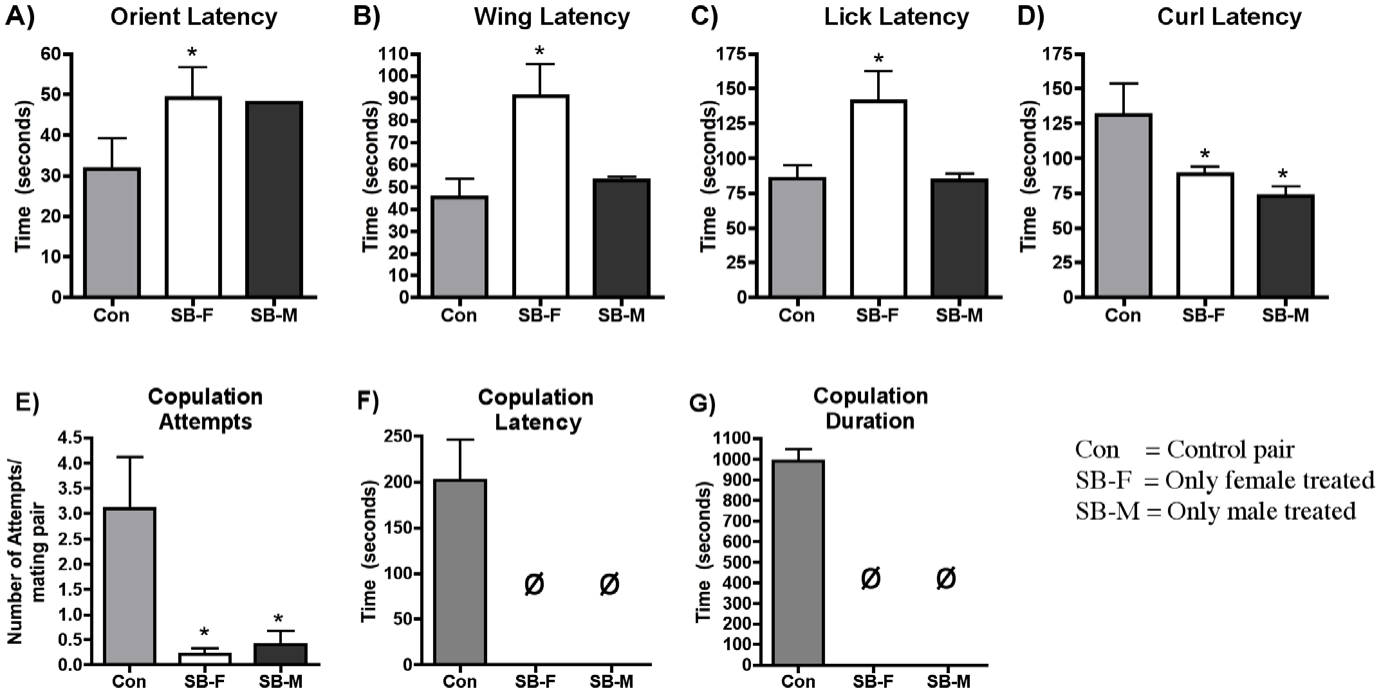
5-HT_7_Dro influences mating behaviors in both male and female Drosophila. Courtship rituals were observed in pairs where either the female (SB-F, white bars) or male (SB-M, black bars) were fed 3mM of the antagonist SB285719 and paired with an untreated partner. Pairs consisting of two untreated flies were used as controls (Gray bars). The time it took for the pairs to perform the courtship behaviors and copulation, the number of copulation attempts and the duration of copulations were measured. In pairs with SB-treated females and untreated males, orient, wing vibration and lick latency (A, B, C) were increased while curl latency (D) was decreased. Pairs with an SB-treated male and untreated male differed significantly only in curl latency (D). The number of copulation attempts was significantly decreased for both experimental sets and neither exhibited any successful copulation (F). Frequencies of courtship behaviors and copulations are listed in Table 1. (n = 10 pairs observed for each behavior; *p<0.01, ANOVA with Bonferroni post hoc test for multiple comparison; φ = No successful copulation)

To determine if the observed impairments in mating behavior in SB treated flies were due to dysfunction in the male, the female, or both, SB fed females were paired with control males, and SB fed males and control females were paired. We chose a dose of 3.0 mM to test, because this dose produced a maximal effect across all behaviors. In the SB female and control male pairs, orient, wing vibration, and lick, latencies were significantly increased (Figures 7 A–4C). Interestingly, curl latencies were significantly reduced (Figure 7D). Copulation attempts (Figure 7E) were also significantly reduced in these pairs. The frequencies at which these behaviors were observed decreased as well compared to control (Table 1). Whereas orient and wing vibration frequencies were only slightly decreased, curl and copulation frequencies were dramatically reduced or eliminated.

**Table 1:** The frequency of courtship behaviors between SB-treated flies and untreated flies.

Behavior	Control	SB-F	SB-M
Orientation	100%	90%	50%
Wing Vibration	100%	80%	20% *
Licking	100%	70%	20% *
Curling	100%	20% *	20% *
Successful Copulations	100%	0% *	0% *

The frequency of courtship behaviors and successful copulations was measured in pairs where either the male (SB–M) or female (SB–F) was fed 3.0 mM of the antagonist and paired with an untreated partner. The frequency of all early courtship behaviors (orient and wing vibrations) was reduced only slightly, but later behaviors (licking, curling and copulation attempts) was significantly decreased in these pairs when compared to untreated control flies. In both experimental sets, successful copulation was never observed. (n = 10 observed pairs for each behavior, *p<0.001 by Fischer's Exact test).

In experiments paring SB males and control females, orient, wing vibration, and lick latencies were not statistically different from control pairs. Curl latency was significantly reduced (Figure 7D), and copulation attempts were significantly and dramatically reduced (Figure 7E). These pairings, as with the SB female + control pairings, did not successfully copulate (Figures 7F–G). Together, these data indicate that 5-HT_7_ receptors may serve to modulate courtship and mating behaviors in both male and females flies. There were some differences observed between these experiments where only one fly was fed SB, and those where both the male and female have been maintained on drug. Specifically, the performance of the SB male flies in the SB male + control were reduced compared to the results of pairs where both flies were exposed to drug. This could be due to differences in the dynamics of the interactions between the male and female when only one partner has been exposed to drug. For example, when both partners have been exposed, the general lack of interest in courtship parallel each other, but there was no active avoidance of one fly from the other. In contrast, when only the males have been exposed, the males were never interested in the females, and tended to stay clear of the females, and generally were never in the same vicinity of the females. Furthermore, there were some instances where the females seemed to seek the male and the male would run away from the female. When only the female was exposed to drug, the male vigorously attempted to court. Whereas these female did not exhibit overtly obvious actions to reject the male, such us wing flicking, they generally avoided contact with the male.

Serotonin has previously been implicated in courtship behaviors, specifically male-male courtship. For example, ectopic expression of the *white (w^+^)* gene, which encodes for the transporter for tryptophan (the metabolic precursor for serotonin) has been shown to lead to inter-male courtship [44], [45]. To determine if SB treatment affected male-male courtship, in addition to normal male-female courtship, we examined interactions between pairs of males fed SB. Control pairs only exhibited minimal male-male courtship behaviors (Figure S2). No significant changes in the courtship index (CI; the time engaged in all courtship behaviors divided by the total time of the assay) in SB-treated males in comparison to untreated controls were detected (Figure S3).

### General olfaction is not altered by drug treatment

We did observe moderate expression of 5-HT_7_Dro-GAL4 GFP in the antennal lobes of the adult brain. To ensure that the deficits in courtship and mating induced by SB treatment were not simply due to general problems with olfaction induced by the receptor antagonist, flies were checked for olfactory avoidance and sensitivity using different concentrations of the odors methylcyclohexanol (MCH) and benzaldehyde (BA). The SB treated flies had performance indices equivalent to control flies at all concentrations of odor, indicating that SB258719 does not impair general olfaction, or sensitivity to odors (Table 2).

**Table 2:** The 5-HT_7_ specific antagonist SB258719 does not affect olfaction.

MCH	Control PI	SB PI
1∶1000	47±2	41±1
1∶750	49±3	47±2
1∶250	59±7	55±1
1∶100	83±3	79±4
BA		
1∶1000	42±3	41±1
1∶750	43±4	45±7
1∶250	47±3	47±5
1∶100	71±4	71±3

Untreated and SB-treated (3.0 mM) flies were assayed for olfactory avoidance and sensitivity at different concentrations of the odors methylcyclohexanhol (MCH) and benzaldehyde (BA). The performance indices (PI) of the SB-treated flies are equivalent to control flies at all concentrations.

### The effects of genetic knock-down of 5-HT_7_Dro mRNA by RNAi are consistent with the results of pharmacological studies

**Figure 8:**
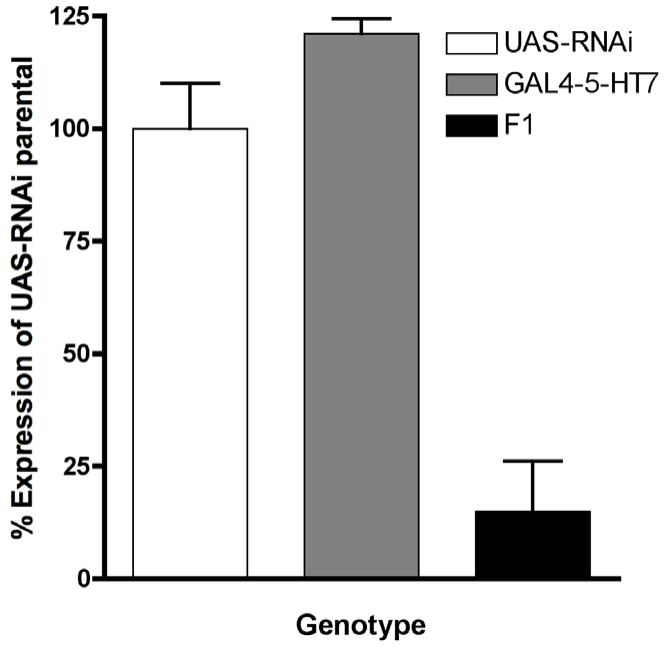
dsRNAi construct effectively reduces 5-HT_7_Dro transcript levels. RNA from the heads of male flies carrying either the sym-p5-HT_7_RNAi (white box), the 5-HT_7_Dro-GAL4 (gray box), or both (F1, black box) transgenes was used in quantitative real-time PCR to examine 5-HT_7_Dro gene expression. Flies carrying both transcripts show an approximately 80% decrease in 5-HT_7_Dro transcript levels. Reactions were performed in quadruplicate for each gene. *RpL32* expression was used as the reference control to normalize expression between treatment groups (Error bars indicate SEM).

Pharmacological and genetic knockdown experiments can provide complementary data regarding the function of a receptor. With pharmacological studies we have measured the effect of different levels of inactivation using different doses of antagonist to show that 5-HT_7_Dro receptor function is potentially required more at the later stages of courtship than the earlier stages. In order to validate our pharmacological results, we created a UAS-dsRNA strain to knock down 5-HT_7_Dro mRNA expression. Quantitative RT-PCR was performed to examine the levels of 5-HT_7_Dro transcript in F1 flies carrying both the 5-HT_7_Dro-GAL4 and the UAS-sym-p5-HT_7_RNAi. Flies carrying both transgenes show an approximately 80% decrease in transcript levels when compared to flies carrying only one trangene (Figure 8).

**Figure 9:**
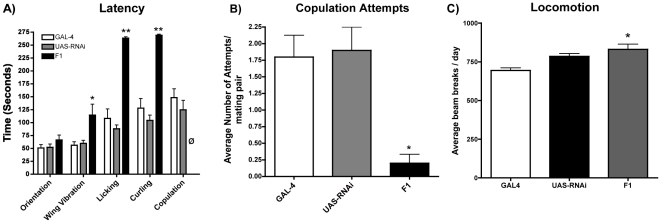
5-HT_7_Dro knock-down results are consistent with pharmacological studies. A) The average latencies of courtship behaviors for flies that performed those behaviors are shown. Transgenic F1 lines expressing 5-HT_7_Dro double stranded RNA under the control of the 5-HT_7_ Dro-GAL4 promoter (black bars) exhibit increased licking and curling latencies, and did not copulate (φ = no successful copulation; * p<0.01; **p<0.001; two-way ANOVA with Bonferroni post hoc analysis). B) The average number of copulation attempts per mating pair are significantly decreased in the F1 knockdowns compared to the parental strains (n = 10 pairs observed per behavior; * p<0.01; one-way ANOVA with Tukey post hoc analysis). C) The activity of male flies carrying either the 5-HT_7_Dro-GAL4 element (GAL4, white bar), the UAS-sym-p5-HT_7_RNAi element (UAS-RNAi, black bar), or both (F1, gray bar) was measured using the DAMS system for five days. The average daily count of beam breaks per 24 hours is slightly increased in the F1 flies with respect to the GAL4 driver parental strain (*), but there is no significant increase in activity when compared with the UAS-RNAi parental. (n = 16 flies; * p<0.05; one-way ANOVA with Tukey post hoc analysis).

If the pharmacological agents (e.g. the SB antagonists) were specifically acting at 5-HT_7_Dro receptors, as anticipated, then knockdown of 5-HT_7_Dro mRNA in putative 5-HT_7_Dro expressing cells would be anticipated to recapitulate major aspects of the behavioral phenotypes of the antagonist. Indeed, expression of double-stranded 5-HT_7_Dro mRNA in cells defined by our 5-HT_7_Dro-GAL4 driver produced courting and mating deficits consistent with the antagonist studies (Figure 9). Early behaviors like orienting and wing vibration were not significantly affected, whereas later behaviors like curling and copulation attempts were significantly reduced, with later events like successful copulation events even eliminated (Figure 9, Table 3). To ensure that these decreases in courtship were not due to decreased activity, the activity levels of flies carrying either one or both of the transgenes were measured. The F1 flies with both transgenes were found to be slightly more active than the 5-HT_7_Dro-GAL4 insertion strain, but not the ds-RNA-UAS parental (Figure 9C).

**Table 3:** 5-HT_7_Dro knock-down decreases frequency of courtship behaviors.

Behavior	Gal4	UAS	F1
Orientation	100%	100%	90%
Wing Vibration	100%	100%	60%
Licking	100%	100%	20% *
Curling	100%	100%	20% *
Successful Copulations	100%	100%	0% *

The frequency of courtship behaviors was measured in transgenic F1 lines expressing the 5-HT_7_-dsRNA under control of the 5-HT_7_-gal4 promoter (F1). When compared with the parental lines 5-HT_7_-Gal4 (Gal4) and UAS-sym-5-HT_7_-dsRNA (UAS), the frequency of licking, curling and copulation attempts was significantly decreased. In the F1 lines, flies were not observed to successfully copulate. (n = 10 observed pairs for each behavior, p<0.001 by Fischer's Exact test).

If either the GAL4 driver strain was not a valid representation of 5-HT_7_Dro expression, or the SB antagonist was not acting at this receptor to inhibit courtship and mating, or the RNAi studies were producing their effects through off target effects or not producing efficient knockdown, expression of 5-HT_7_Dro double stranded RNA to produce RNA interference within the circuits defined by our 5-HT_7_Dro-GAL4 driver would not produce behavioral effects consistent with the pharmacological studies. Therefore, we believe that these methods cross validate each other, as well as the driver strain, to demonstrate that we are indeed examining 5-HT_7_Dro receptor function within the brain.

## Discussion

To explore the role of the 5-HT_7_Dro receptor in the fly, we have created an enhancer GAL4 driver strain and used it to characterized the putative CNS expression and function of the receptor. Using our 5-HT_7_Dro-GAL4 driver to drive expression of a UAS-mCD8::GFP transgene, we have found GFP expression in third larval instar brain localized to discreet circuits within the brain hemispheres, as well as to specific neurons in the ventral ganglion. In the adult brain, there is a high level of 5-HT_7_Dro-GAL4 expression in large-field R neurons that innervate the ellipsoid body, as well as in neurons in the brain that tightly cluster with the PDF-positive LNv clock neurons and innervate the optic lobes. There is moderate expression detected in the olfactory and gustatory regions of the brain, and weak expression in other central complex structures like the fan shaped body. 5-HT_7_Dro-GAL4 expression appears to be post-synaptic both in larvae and adults. This is consistent with the observed post-synaptic expression for the 5-HT_7_ receptor in vertebrate CNS [4]. In the ventral nerve cord, expression is detected in several sets of postsynaptic neurons and in all neuromeres of the fused ganglion. Importantly, there is a close apposition between receptor-expressing processes and those containing serotonin. Although a majority of GAL4 driver strains present relevant/accurate expression data of the intended gene [46], It should be emphasized here that 5-HT_7_Dro-GAL4 enhancer expression may not represent the entire expression pattern of the native 5-HT_7_Dro receptor, or may be even expressing in cells that do not express the native receptor. It should also be emphasized that the mCD8:GFP construct we used in this study to examine expression is a membrane-bound form of the GFP protein that highlights all cellular membranes. Whereas the GFP expression patterns observed here provide clues as to the cells and structures that express the 5-HT_7_Dro-GAL4 element, it is unable to provide information regarding the subcellular localization of the GPCR on the cellular membrane. Unfortunately, our attempts to generate anti-sera to 5-HT_7_Dro for further validation of native protein expression were unsuccessful.

Treatment with the 5-HT_7_ receptor antagonist SB258719 interferes with courtship and mating behaviors. Interestingly, the IC_50_ values for inhibition of courtship behavior frequency decreases as the courtship and mating process progresses from hardly any disruptive effect with respect to early behaviors involving sensory cues (orient and wing vibration), to more pronounced effects for intermediate behaviors (licking, curling, attempts), to complete loss of successful copulation at higher levels of the drug. In corroboration with the pharmacological studies, knockdown of 5-HT_7_Dro message within 5-HT_7_Dro-GAL4 expressing neurons produces behavioral changes consistent with our pharmacological results. The early behaviors of orientation and wing song are not affected much, the intermediate behaviors of licking and curling are significantly disrupted, and successful copulation is eliminated. There are some subtle differences between methods, however, like for curling latencies, which could be due to the nature of receptor inactivation (more acute pharmacological methods *vs*. constitutive nature of the RNAi knockdown).

From our results, there appears to be little 5-HT_7_Dro receptor involvement in the early stages, where we observed that antagonist treated males are receptive to females present in the mating chamber, and are able to initiate the first elements of the mating process. During the intermediate stages, involving licking and curling, there is likely more involvement of 5-HT_7_Dro receptor signaling. A decrease in licking behavior may result in decreased curling, and decreased curling may result in decreased successful copulation attempts, with the effects compounding at each successive behavior leading to an overall failure at successful copulation. These effects could arise from alterations in physical performance and coordination, sensory perception, olfaction, or to decreases in receptivity or interest, or a lack of ‘motivation’ to perform the higher intensity physical behaviors. We believe these effects are not due to deficits in overt locomotor activity because the drug treated flies demonstrate normal levels of measured activity, or in coordination because at drug levels where mating frequency is decreased the flies that do perform, perform well with latencies not significantly different than control pairs. General alteration of sensory perception is also not a likely reason because males recognize females and readily perform behaviors related to visual and acoustic cues (orienting and wing vibration) despite administration of antagonist or knockdown of message. Whereas olfaction is necessary for receptivity, it is unlikely impeded following antagonist administration because flies fed 3 mM SB as well as the knockdown flies exhibit normal aversion and sensitivity to odors in olfactory tests. Nevertheless, there still may be an olfactory or pheromone component if the limited 5-HT_7_Dro-GAL4 expression detected in the olfactory lobes correlates with select neurons necessary for reception of specific courtship related pheromones, or the expression does not correlate appropriately with native 5-HT_7_Dro expression and localization in the olfactory lobes.

A key gene known to be significantly involved in courtship behaviors is *fruitless* (*fru*), where almost every stage of the mating process has been shown to be disrupted by certain alleles of the locus [47]. With the exception of one *fru* allele, *satori,* where males exclusively courts males, *fru* mutants indiscriminately court both males and females [48], [49], [50]. In Drosophila, there are also several other known mutants that rarely court females or males. These include *he's not interested* (*hni*), *tapered* (*ta*), *pale*, *cuckold* (*cuc*) and *courtless* (*crl*) [51]. Two of these genes have been characterized: *courtless* encodes a ubiquitin-conjugating enzyme that is also involved in spermatogenesis [52], and *pale* encodes a tyrosine hydroxylase that catalyzes the synthesis of dopamine [53]. In our experiments with both the 5-HT_7_ receptor antagonist and the RNAi knockdown, male flies displayed a general disinterest in mating with either females or other males, and females appeared non-responsive to male courtship attempts.

Ectopic expression of the *white* gene, which encodes for the transporter for the biosynthetic precursor of serotonin, tryptophan, can induce inter-male courtship [44], [45]. These observations, along with others, suggested that serotonin may be involved in courtship in the fly (interestingly, alterations in 5-HT levels have been shown to elicit homosexual behavior in mammals [54], [55]). In early studies examining co-expression of the male-specific Fru^M^ protein and 5-HT, it was found that in wild-type males there were no 5-HT CNS neurons that co-expressed Fru^M^, with the exception of a small cluster of serotonergic cells at the posterior tip of the ventral nerve cord [37]. The possibility, however, still remained that perhaps Fru^M^ expression was mediating the effects of serotonin postsynaptically. Our results have shown there is no overlap between Fru^M^ and 5-HT_7_Dro-GAL4 expression. Therefore, it would appear that 5-HT_7_Dro and Fru^M^ do not directly interact in their modulation and control of courtship and mating behaviors.

What then is the role that 5-HT_7_ receptor signaling plays in reducing receptivity at each stage? One possibility is that pheromone release from the female may be disrupted, resulting in a decrease of courtship. We believe that this is unlikely, however, because control males paired with SB fed females continue to attempt copulation with SB fed females that are not receptive, and will continually chase these females in the mating chamber, which remain uninterested despite repeated attempts by the males. Furthermore, when males fed SB were paired with control females, we often observed females that seemed to seek out the male, which would then run away from the female. Therefore, it would appear that because the control fly of the pair seeks out and attempts to initiate courtship behaviors, SB treatment and manipulation of 5-HT_7_ receptor function does not interfere with pheromone release, or potentially other sensory cues, related to receptivity. If the default behavior is to initiate courtship in the absence of pheromones, however, then it may still be possible that SB is interfering with pheromone reception at later stages if they are required for the maintenance and intensification of courtship. In the SB/control pairing experiments, the SB fed male + control female pairs were the least successful at courtship behavior. One interpretation of these results are that males are essential to initiating certain elements of mating that are regulated by 5-HT_7_ receptor function, and when these do not occur it may contribute to further lack of receptivity by females. Alternatively, males and females may be only responding to the SB drug differently.

Because of the high level of expression of the 5-HT_7_Dro-GAL4 driver in the large-field R field neurons that innervate the ellipsoid body, we hypothesize that receptor expression in these neurons is relevant for normal courtship and mating. Furthermore, that the 5-HT_7_ receptors expressed by these neurons regulate receptivity/interest of one fly for its partner. Little is known regarding the exact function of the ellipsoid body, and the neurons that feed into it. The overall structure is believed to be involved in mediating higher order behaviors, including aspects of learning and memory [56], [57], [58], stress response [59], flight control [60], and gravitaxis [61]. It is comprised of many distinct cell types and individual circuits including 10 types of small field neurons, and 4 known major types of large-field R neurons [31]. Previous reports have indicated that there are about 40 large-field R neurons in each of two clusters in the central brain [31], and based upon the number of large-field R neurons that we detect 5-HT_7_Dro-GAL4 GFP expression in, there may be nearly 50, suggesting that there are additional subtypes of this neuron beyond those already identified. Significantly, 5-HT_7_Dro-GAL4 may be the first GAL4 driver line reported that predominantly and strongly drives expression in the putative full set of large-field R neurons within the central brain, and as such may be a valuable strain to use to define the functional role of these cells and the development of ellipsoid body circuits. A subset, the R4m neurons, are cholinergic and express NMDA receptors [58]. It remains to be seen which of the major subsets of large-field R neurons contribute to courtship and mating behaviors. Although we hypothesize that it is the ellipsoid body mediating courtship behaviors, it is entirely possible that other neurons that are weakly expressing 5-HT_7_Dro-GAL4, or neurons perhaps not expressing the 5-HT_7_Dro-GAL4 element in the brain, are contributing to or completely mediating these observed effect on courtship and mating.

Courtship and mating has been shown by others to involve additional neurotransmitter systems including dopamine, which has been shown to play a role in both female receptivity [24] as well as male-male courtship [62], [63], and GABA, which also plays a role in female receptivity [64]. Various brain structures including the mushroom bodies, protocerebrum, and optic lobes [47] have also been shown to be involved in courtship and mating in the fly. With regards to courtship and mating, the 5-HT_7_Dro circuitry, the ellipsoid body, or both, may be essential to the integration and processing of information received from the various sensory stimuli to correctly initiate physical courtship behaviors. Significantly, proper function of Gαs-coupled 5-HT_7_Dro receptor signaling within these neurons may also be essential to this process. Interestingly, 5-HT_7_ receptors have also been implicated in the regulation of mammalian sexual behavior, although the response is reverse to that in flies. Blockade of this receptor with antagonist in female rats increases lordosis activity, and the authors of the study hypothesize that in mammals 5-HT_7_ receptors exert a tonic inhibitory effect on female sexual behavior [15].

In summary, we have generated a 5-HT_7_Dro-GAL4 reporter and have used it to characterize the putative expression of the 5-HT_7_Dro receptor, and find it highly expressed in the brain in large-field R neurons in the adult, as well as in small groups of cells that cluster with PDF-positive LNvs neurons. Functional studies utilizing pharmacological and genetic methods indicate that this receptor is necessary for normal courtship and mating.

## Supporting Information

Figure S1 **5-HT_7_Dro-GAL4 expression in relation to serotonin-immunoreactive neurons**. Ai and Aii. The cell bodies (cb) of the R-neurons express the reporter, but not serotonin (at arrow). Bii and Bii. The large serotonergic neurons of the antennal lobes (arrows in Bii) do not express the reporter. C. One neuron in each antennal lobe displays 5-HT_7_Dro-GAL4 expression (arrow). Di and Dii. This neuron does not produce serotonin, as seen in this double labeling. AL, antennal lobe.
(TIF)

Figure S2 **Distribution of male form of Fruitless (Fru^M^) and 5-HT_7_Dro-GAL4 expression in adult ventral nerve cord**. Gal4-expression is shown in green and Fru^M^ imunolabeling in magenta in these horizontal views of the ganglion (anterior is up in all panels and the scale bar applies to all panels, except 5iv which is a slight enlargement). Ai-iii) No colocalization of Fru^M^ and GFP in neurons of the pro- and mesothoracic neuromeres. Bi-iv) No colocalization of markers in meso- and metathoracic neuromeres. Some neurons appear white (in boxed area) due to superposition in this projection of about 12 sections. In panel Biv we show two optical sections in the boxed area to visualize that neurons do not coexpress markers. C) Also in abdominal neuromeres the two labels are not coexpressed.
(TIF)

Figure S3 **SB-treated male flies do not exhibit increased intermale courtship.** The Courtship Index (CI) was measured for untreated male/female pairs (white), untreated male/male pairs (gray) and SB treated males/male pairs (Black). The CI of male/female pairs was 0.75, consistent with published reports. The CI for untreated male/male pairs was less than 0.1. Male pairs treated with 3 mM SB did not show a significant increase in intermale courtship when compared to untreated male/male pairs. (Error bars =  SEM; *p = <0.01 vs m/f; ANOVA with Tukey's Multiple Comparison Test).
(TIF)
