# Supplementary figures and images for: The Serotonin 5-HT7Dro Receptor Is Expressed in the Brain of Drosophila, and Is Essential for Normal Courtship and Mating
Source: PLoS One. 2011 Jun 2;6(6):e20800. doi: 10.1371/journal.pone.0020800 (PMC3107233; doi:10.1371/journal.pone.0020800)

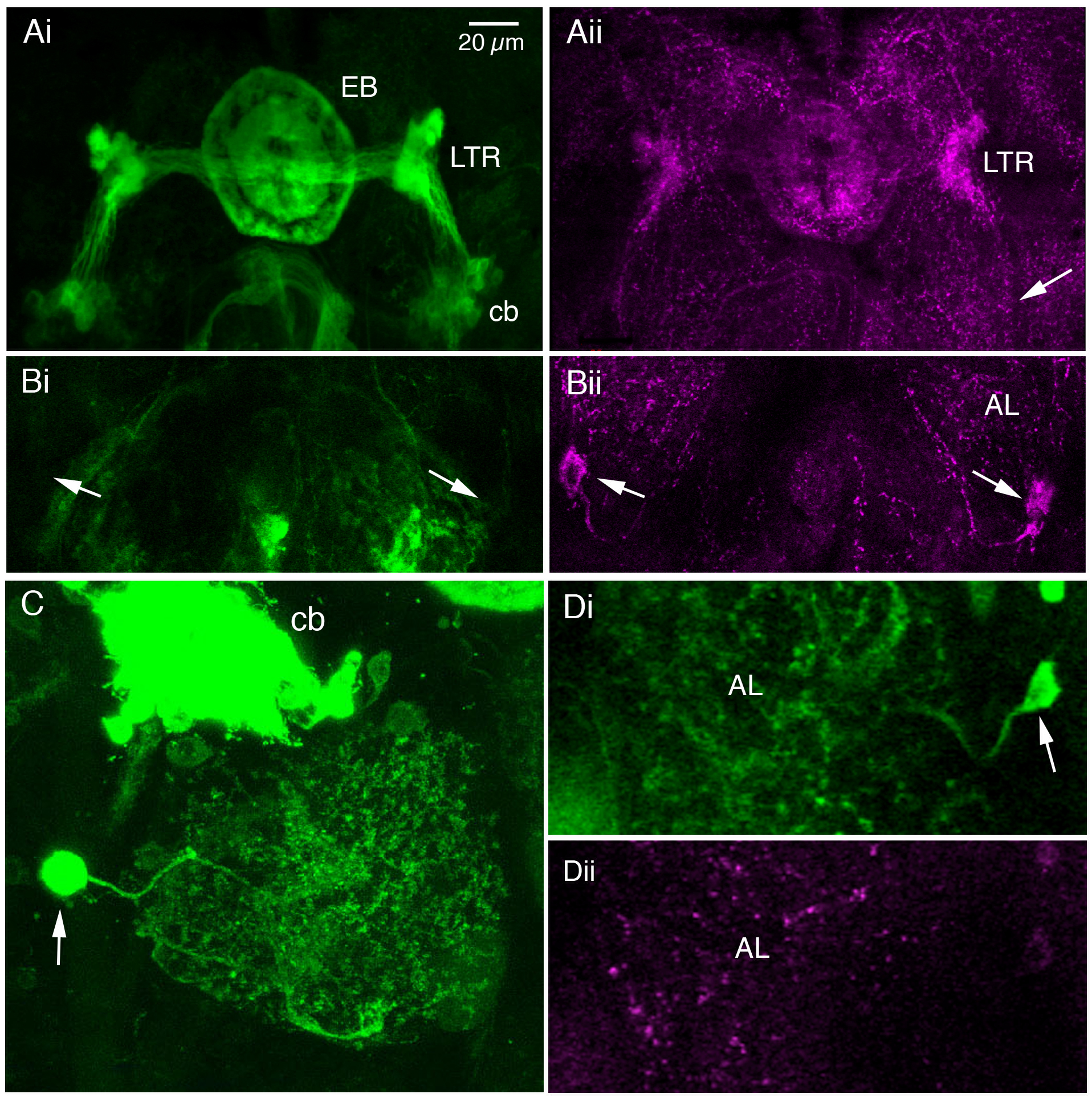

Supplement: Figure S1 — 5-HT7Dro-GAL4 expression in relation to serotonin-immunoreactive neurons. Ai and Aii. The cell bodies (cb) of the R-neurons express the reporter, but not serotonin (at arrow). Bii and Bii. The large serotonergic neurons of the antennal lobes (arrows in Bii) do not express the reporter. C. One neuron in each antennal lobe displays 5-HT7Dro-GAL4 expression (arrow). Di and Dii. This neuron does not produce serotonin, as seen in this double labeling. AL, antennal lobe. (TIF) [file pone.0020800.s001.tif]

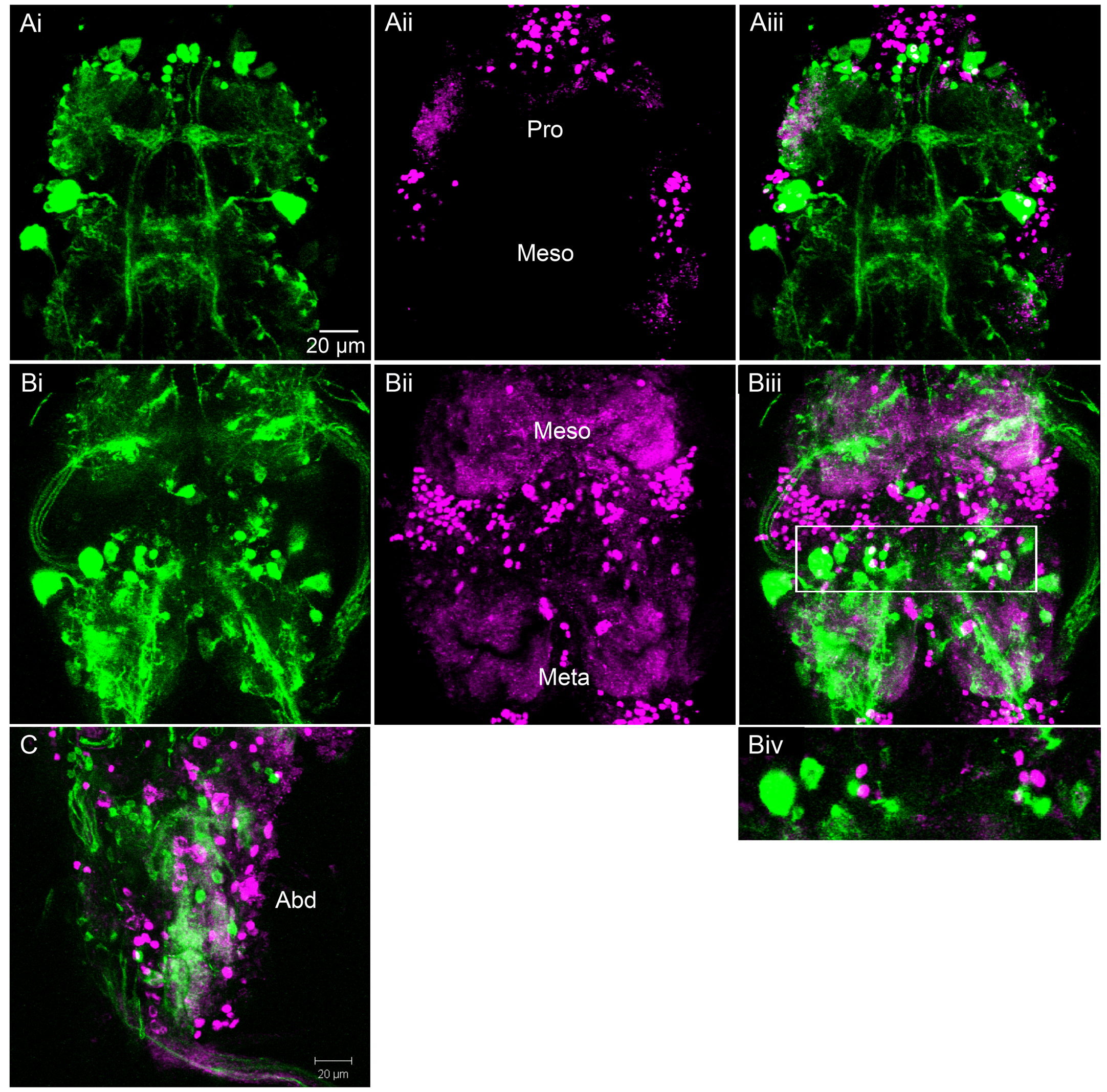

Supplement: Figure S2 — Distribution of male form of Fruitless (FruM) and 5-HT7Dro-GAL4 expression in adult ventral nerve cord. Gal4-expression is shown in green and FruM imunolabeling in magenta in these horizontal views of the ganglion (anterior is up in all panels and the scale bar applies to all panels, except 5iv which is a slight enlargement). Ai-iii) No colocalization of FruM and GFP in neurons of the pro- and mesothoracic neuromeres. Bi-iv) No colocalization of markers in meso- and metathoracic neuromeres. Some neurons appear white (in boxed area) due to superposition in this projection of about 12 sections. In panel Biv we show two optical sections in the boxed area to visualize that neurons do not coexpress markers. C) Also in abdominal neuromeres the two labels are not coexpressed. (TIF) [file pone.0020800.s002.tif]

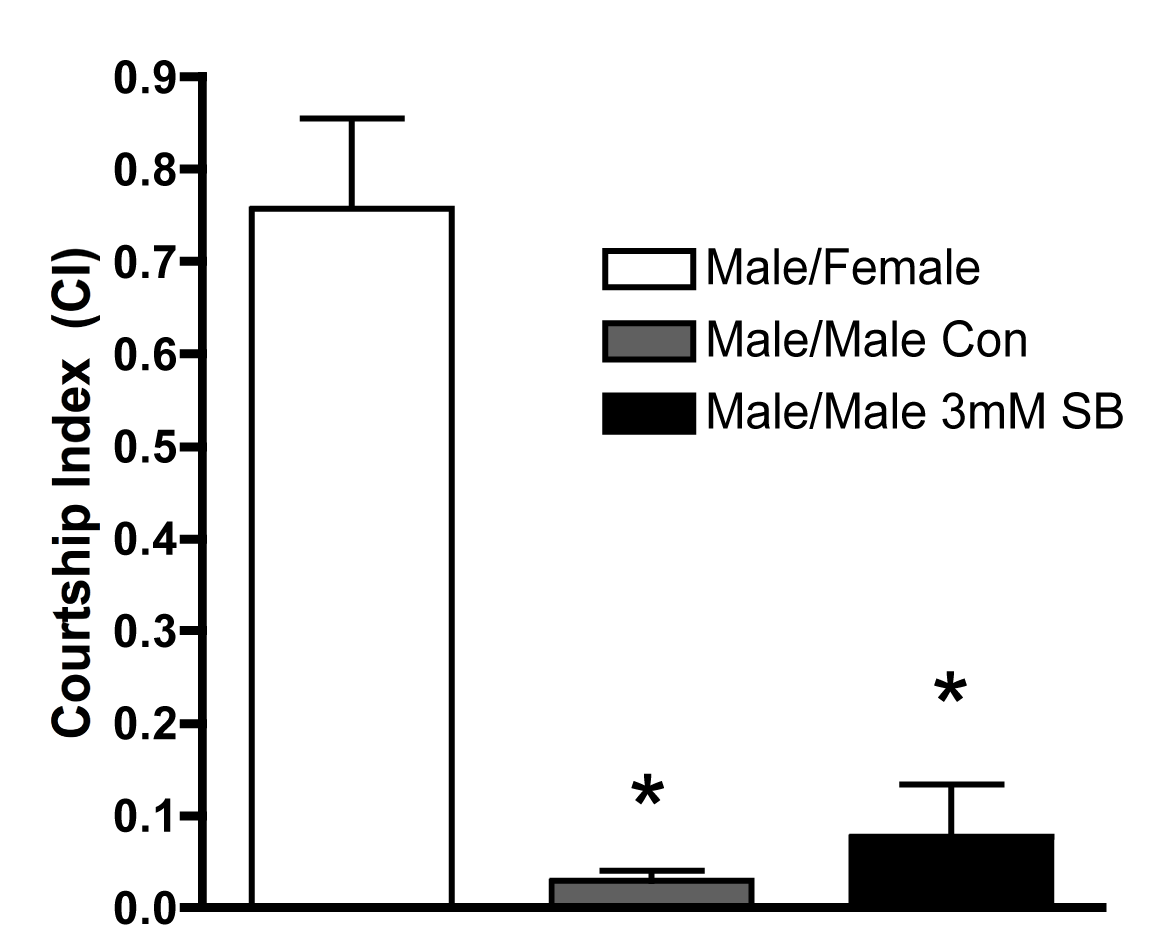

Supplement: Figure S3 — SB-treated male flies do not exhibit increased intermale courtship. The Courtship Index (CI) was measured for untreated male/female pairs (white), untreated male/male pairs (gray) and SB treated males/male pairs (Black). The CI of male/female pairs was 0.75, consistent with published reports. The CI for untreated male/male pairs was less than 0.1. Male pairs treated with 3 mM SB did not show a significant increase in intermale courtship when compared to untreated male/male pairs. (Error bars = SEM; *p = <0.01 vs m/f; ANOVA with Tukey's Multiple Comparison Test). (TIF) [file pone.0020800.s003.tif]
